# Pseudokidney Sign in Gastric Cancer

**DOI:** 10.3390/diagnostics14090896

**Published:** 2024-04-25

**Authors:** Thomas Ferenc, Jelena Svetec Dvorski, Andro Matković, Maja Mijić, Eva Lovrić, Vinko Vidjak

**Affiliations:** 1 Department of Diagnostic and Interventional Radiology, Merkur University Hospital, 10000 Zagreb, Croatiaandro.matkovic@gmail.com (A.M.);; 2 Department of Internal Medicine, Division of Gastroenterology, Merkur University Hospital, 10000 Zagreb, Croatia; 3 Department of Pathology, Merkur University Hospital, 10000 Zagreb, Croatia; 4 School of Medicine, University of Zagreb, 10000 Zagreb, Croatia

**Keywords:** stomach neoplasms, ultrasonography, pseudokidney sign

## Abstract

Pseudokidney sign (PKS) is a characteristic sonographic finding of an abnormal mass with a reniform appearance, and a hyperechoic central region surrounded by a hypoechoic area. It has been seldom documented in gastric cancer. A 75-year-old male patient presented with a palpable abdominal resistance in the left upper abdominal quadrant and ultrasound evaluation revealed a well-vascularized mass presenting with PKS. Regional lymphadenopathy was also found, and the working diagnosis of gastric cancer was established. The suspected diagnosis was later verified endoscopically and on pathohistological examinations as gastric adenocarcinoma. Computed tomography staging also revealed distant metastases to the lungs, liver, and adrenal glands and abdominal lymphadenopathy. The PKS often indicates gastrointestinal pathology, and it may be seen in benign and malignant conditions due to gastrointestinal wall thickening. Therefore, additional diagnostic examinations are advised for a more definite diagnosis.

Gastric cancer (GC) is a global public health problem. It is the fifth most common malignancy and the fourth leading cause of cancer-related deaths among all cancer types in the world (following lung, colorectal, and liver cancer) [1]. It was estimated that around 769,000 annual deaths were closely related to GC, with males having twice the risk of the disease in comparison to females [1]. Incidence rates are the highest in Eastern Asia and Eastern Europe, whereas incidence rates in Northern America and Northern Europe are mostly low and similar to those detected in Africa [2]. 

Pseudokidney sign (PKS) is a characteristic sonographic finding of an abnormal mass resembling a normal kidney (Figure 1) with a reniform appearance and a hyperechoic center surrounded by a hypoechoic area [3,4]. The sign was first reported in colorectal carcinoma [4], and since then it has been documented in many other bowel disorders such as intussusception, necrotizing enterocolitis, midgut volvulus, sigmoid volvulus, diverticulitis, regional enteritis, small bowel lymphoma, and small bowel ischemia [3,4,5,6]. However, the sign is not pathognomonic for bowel pathology because it has been seldom reported in gastric cancer [4,6]. According to Mumoli et al. [4], gastric cancer, particularly a large fungating type with eccentrically located gastric lumen and perigastric infiltration, can lead to a segmentally thickened and edematous gastric wall. Sonographically, the edematous wall is hypoechoic, and the central hyperechoic region is created by mesentery and food, resulting in PKS. Therefore, any gastrointestinal disease that includes circumscribed and hypoechoic wall thickening around the hyperechoic lumen may lead to sonographic findings of PKS [4].

A 75-year-old male patient presented in the emergency department with a 2-month history of dull pain in the epigastric region, followed by loss of appetite, multiple episodes of postprandial vomiting, and 10 kg weight loss during that period. The patient also consumed a small amount of alcohol daily for years and had a 50-pack-year smoking history. Otherwise, his medical record had no active medical treatments or serious illnesses. During clinical examination, the main finding was palpable abdominal resistance in the left upper abdominal quadrant. After laboratory blood and urine sampling, the patient was referred to a transabdominal ultrasound (US). The US demonstrated a well-vascularized mass in the left upper abdominal quadrant, measuring 4.1 × 10.1 × 9.4 cm, presenting with a reniform appearance and hyperechoic center surrounded by a thick hypoechoic wall. The described findings were characteristic of PKS (Figure 2). Regional lymphadenopathy was also detected, and the working diagnosis of gastric cancer was established. 

The patient was then referred to an endoscopic examination which found the infiltrative tumor in the gastric body, non-passable for the instrument (Figure 3). Several tissue samples were collected and the pathohistological analysis confirmed the diagnosis of gastric adenocarcinoma (Figure 4). Standard contrast-enhanced chest–abdomen–pelvis computed tomography (CT) staging was performed and it revealed distant metastases to the lungs, liver, and adrenal glands, and abdominal lymphadenopathy. To decrease his tumor cachexia, surgeons formed a feeding jejunostomy in the upper left abdominal quadrant. Due to the extensive metastatic spread of the disease, the patient was sent to the institution for palliative care. 

The presence of PKS is an indirect sign of possible bowel pathology that requires medical attention and early recognition is essential; however, the sign is not pathognomonic, and it may be present in benign or malignant conditions (e.g., gastric linitis plastica, Menetrier disease) due to gastrointestinal wall thickening [6]. Therefore, additional diagnostic procedures such as endoscopy or CT are advised to confirm the suspected diagnosis.

## Figures and Tables

**Figure 1:**
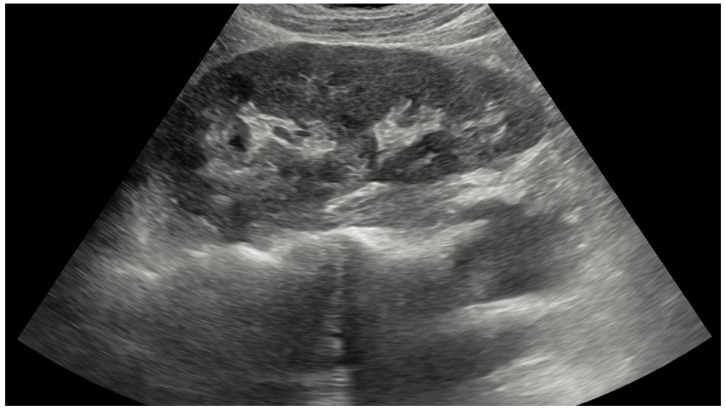
Ultrasound image of normal kidney with centrally located hyperechoic renal sinus surrounded by hypoechoic renal parenchyma.

**Figure 2:**
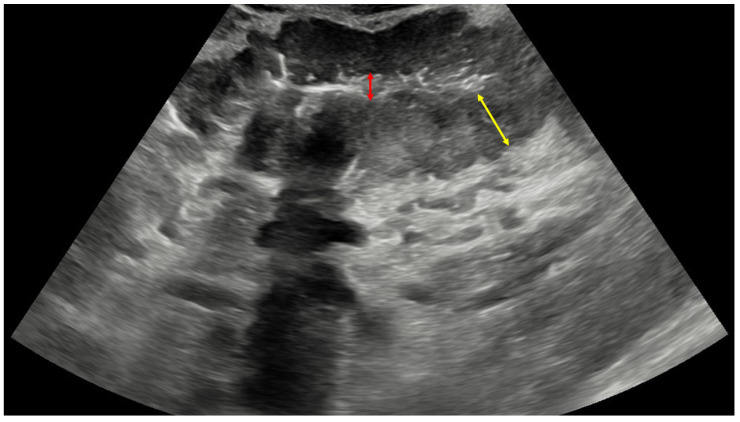
Ultrasound image of pseudokidney sign in this patient with the centrally located hyperechoic gastric lumen (red arrow) surrounded by hypoechoic and thickened gastric wall (yellow arrow), indicative of gastric cancer. Additional findings that may be present in such cases are hypervascularity of the mass, enlarged lymph nodes, and hyperechoic surrounding mesenteric fat.

**Figure 3:**
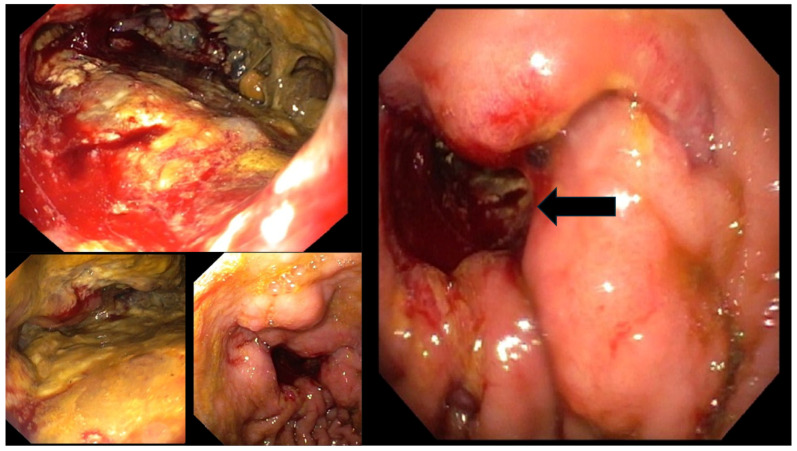
Esophagogastroduodenoscopic images of this patient. The esophageal mucosa appeared normal. Upon stomach intubation, significant aspiration was required to evacuate substantial retained gastric contents. In the central portion of the gastric body, an infiltrative neoplasm was identified, characterized by superficial hemorrhage and areas of necrosis. The tumor obstructed the passage of the endoscope distally. The arrow on the right-hand image indicates the site of complete infiltration of the gastric corpus mucosa by the tumor, making it non-passable for the endoscope. Several biopsies were obtained for histopathological examination.

**Figure 4:**
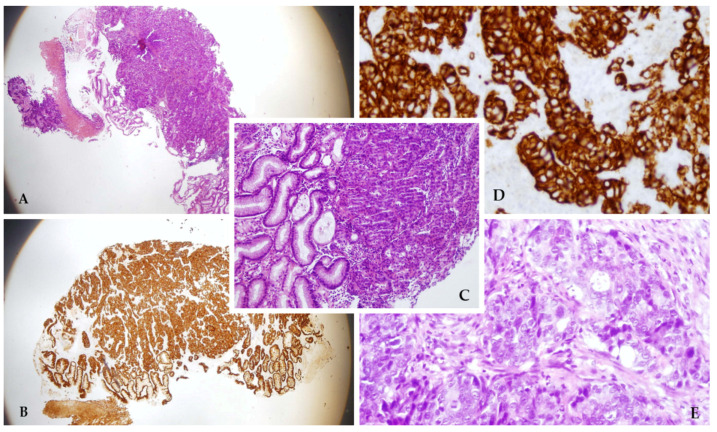
Pathohistological images of endoscopically collected samples ((**A**)—H&E, magnification 4×; (**B**)—immunohistochemistry, 4×; (**C**)—H&E, 10×; (**D**)—immunohistochemistry, 20×; (**E**)—H&E, 20×). Three pieces of tumor tissue measuring up to 2 mm. The tumor comprised clusters and streaks of atypical epithelial cells and prominent nucleoli. Immunohistochemically, the tumor cells were positive for CKAE1/AE3. The stomach lining can be seen on the surface of one sample. It corresponds with gastric adenocarcinoma.

## Data Availability

Data are contained within the article.
